# Malaria survey data and geospatial suitability mapping for understanding spatial and temporal variations of risk across Kenya

**DOI:** 10.1016/j.parepi.2024.e00399

**Published:** 2024-12-14

**Authors:** Caroline Kioko, Justine Blanford

**Affiliations:** ITC Faculty Geo-Information Science and Earth Observation, University of Twente, Enschede, the Netherlands

**Keywords:** Malaria survey data, Geospatial suitability mapping, Spatial, Temporal, Variations, Risk

## Abstract

Malaria remains a public health concern in Kenya where children and pregnant women are vulnerable groups. The common interventions in place to fight malaria include using insecticide-treated bed nets (ITNs), knowledge and awareness about malaria, and intake of malaria anti-malaria drugs. Despite the availability of these interventions, Kenya still records more than 10,000 clinical cases annually. In this study, we examined how malaria and interventions varied across Kenya for 2015 and 2020. We analyzed the Kenya Malaria Indicator Survey (*N* = 10,072) for 2015 and, (*N* = 11,549) for 2020, and climate data with Fuzzy overlay method to examine how malaria and its interventions relate to environmental conditions required for malaria. The study found that 79 % of malaria cases were distributed in lake endemic, 11 % in coastal endemic, 7 % in highland epidemic, and 3 % in seasonal zone. Use of Insecticide-treated bed nets (ITNs) was 77 % in lake endemic, 13 % in coastal endemic, 9 % in highland epidemic, and 1 % in seasonal zone. Knowledge about malaria was 82 % in lake endemic, 9 % in highland epidemic, 6 % in coastal endemic, and 3 % in seasonal zone. Additionally, based on climate data, lake endemic zone was 94 % suitable for malaria transmission compared to other zones. Despite the use of ITNs and awareness about malaria, malaria transmission continues to be a threat especially in counties in the lake endemic zone. Furthermore, place of residence, climate factors, ownership of ITNs may be associated with malaria in the region.

## 1 Introduction

Malaria is an infectious disease caused by *Plasmodium* parasites (Girum et al., 2019) and transmitted to people through the bite of infected female *Anopheles* mosquitoes (WHO, 2022). Despite the use of insecticide-treated bed nets (ITNs) and malaria awareness, malaria remains a global public health threat (Girum et al., 2019) with 249 million cases reported in 85 malaria endemic countries resulting in 608,000 deaths (WHO, 2022). Although, malaria is declining in the Sub-Saharan region, Kenya still records over 3.5 million new clinical cases and 10,000 deaths annually (WHO, 2022).

Malaria Indicator Surveys (MIS) were developed to provide valuable information on malaria control and elimination (KMIS, 2021). These surveys are individual-level surveys conducted at the household level to provide information on the general population rather than on those seeking health care (Guerra et al., 2019) and are done every five years with between 5000 and 30,000 households as the sample size (Croft et al., 2018). MIS uses a two-stage sampling procedure proportional to the population of the entire country as described in (Croft et al., 2018). For the past two decades, these surveys have collected information about malaria prevention and treatment practices, health seeking behavior, knowledge about malaria, malaria morbidity and mortality (Guerra et al., 2019). The data collected from the MIS have been used in several studies (Table 1) to study malaria interventions coverage and treatment practices (Jima et al., 2010), understand health seeking behavior among under-five years old (Nabyonga Orem et al., 2013), knowledge about malaria and prevention among women 15–49 years of age (Hwang et al., 2010), and human mobility and malaria among children (Bradley et al., 2015). These studies found that the majority of households use nets, children living in rural areas seek healthcare more than children living in urban areas, mothers of under five children had malaria knowledge and travelling children are usually at greater risk of getting infected.

**Table 1:** Summary of how MIS has been used to better understand the ecology of malaria and assess intervention practices.

MIS use	Study area	Year	Population	Method	Findings	Reference
Malaria prevention and treatment practices	Ethiopia	2007	children <5 years; women aged 15–49	Descriptive analysis	households owned at least one net and children with fever sought medical attention within 24 h	(Jima et al., 2010)
Health seeking behavior	Uganda	2009	children <5 years	Binomial logit and Log-linear regression models	children in rural areas seek healthcare more than urban areas and private providers are the preferred source of treatment	(Nabyonga Orem et al., 2013)
Knowledge about malaria and prevention	Ethiopia	2007	women aged 15–49	Multivariate logistic models	mothers of under five children from poorest wealth, no education level, living in rural areas had less malaria knowledge	(Hwang et al., 2010)
Human mobility and malaria	Bioko Island, Equatorial Guinea	2013;2014	children aged 2–14; adults	Logistic regression models and random effects models	children who had travelled were at greater risk of infection	(Bradley et al., 2015)

Geographic information system (GIS), statistical and spatial analysis methods have been widely used to study the spatial and temporal distribution of malaria (Table 2) and interventions (Table 3). These have been used to map malaria prevalence (Hay et al., 2009) using clinical information and predict malaria risk using climate variables (Blanford et al., 2013; Craig et al., 1999). For example, (Kioko and Blanford, 2023) used a fuzzy logic method and (Blanford et al., 2013) applied geo computational methods with host-pathogen-environment models to map areas suitable for malaria transmission to occur using temperature and rainfall information in sub-Saharan Africa. (Hay et al., 2009) used Bayesian geostatistical methods with parasite prevalence information to map *Plasmodium falciparum* malaria endemicity while (Giardina et al., 2014) examined the effects of vector-control interventions on changes in risk of malaria *parasitemia* in sub-Saharan Africa. When risk maps are combined with population information, they were useful for assessing populations at risk. For example, in Africa 53 % of population were found to reside in high endemicity areas, 30 % live in intermediate risk areas and 17 % in low stable risk areas (Hay et al., 2009). Lastly, (Warkaw et al., 2022) used Global and Local Moran's I, and auto logistic spatial binary regression model to determine spatial pattern and predictors of malaria in Ethiopia. The study found that house characteristics such as (floor and roof-type) were important in determining malaria risk (Warkaw et al., 2022).

**Table 2:** Summary of how GIS, statistical and spatial methods have been applied for mapping malaria.

Aim	Data used	Geographical location	Method	Finding	Reference
Distribution of malaria transmission	Temperature; rainfall	sub-Saharan Africa	Fuzzy logic model	Suitable zones have high transmission intensity and unsuitable zones have low transmission intensity.	(Craig et al., 1999)
Understanding the past and chart the future of malaria control using spatio-temporal methods	*Plasmodium falciparum* prevalence surveys: children data aged 2–10 years	Kenya	Spatio-temporal geostatistical models	Declining prevalence was not equal across the country and 8.5 % of 2015 population live in areas with *Plasmodium falciparum* parasite prevalence ≥30 % while 61 % live in areas with <1 %.	(Macharia et al., 2018a)
Implication of temperature variation for malaria parasite development	Temperature	Kenya and across Africa	Geo computational methods with host-pathogen-environment models	Under cool conditions, the use of mean monthly temperatures will underestimate parasite development and under warm conditions the use of mean temperatures will overestimate parasite development.	(Blanford et al., 2013)
Mapping *Plasmodium falciparum* malaria prevalence	*Plasmodium falciparum* parasite rate; population data	Global	Bayesian geostatistical methods	Fifty-three percent of those at risk in Africa live in high endemicity areas, 30 % live in intermediate risk areas and 17 % live in low stable risk areas; 90.1 % of people within sub-Saharan Africa continue to live in endemic areas, and the region account for 79.4 % of cases and 87.6 % of deaths in 2017.	(Hay et al., 2009),(Weiss et al., 2019)
Spatial pattern and malaria predictors	Malaria survey data	Ethiopia	Global and Local Moran's I, and auto logistic spatial binary regression model	Malaria risk is higher in soil floor-type houses compared to tiled and other roof-type houses.	(Warkaw et al., 2022)

**Table 3:** Summary of how GIS, statistical and spatial methods have been applied for mapping interventions.

Aim	Data used	Geographical location	Method	Finding	Reference
Assess the effects of malaria interventions on the geographical distribution of parasitaemia risk	Malaria survey data	Burkina Faso	Bayesian geostatistical models	Effect of ITN coverage and ACT coverage on parasitaemia risk was not important at national level, and was important at local level	(Diboulo et al., 2016)
Explore the impact of the intervention coverage and people's adherence to the interventions on malaria health outcome	Village/health facilities locations; ITN coverage and adherence; Malaria RDTs results	Laos	Spatial analysis	Malaria cases were detected in villages, where intervention coverage and adherence to the intervention remained lower.	(Shirayama et al., 2009)
Assess the effects of vector-control interventions on changes in risk of malaria parasitemia	Malaria data; climatic data	sub-Saharan Africa	Bayesian geostatistical models	Insecticide-treated bed nets and indoor residual spraying are effective in controlling malaria transmission.	(Giardina et al., 2014)
Examine healthcare utilization for treatment of fever among children below 5 years	Healthcare facilities; population; land cover; auxillary data	Northern Namibia	Three parameter logistic model, cost allocation tools	Children living near the health facility sought fever treatment faster compared to those living one hour away from the facility.	(Alegana et al., 2012)

Kenya's aim is to reduce malaria incidence and mortality by 75 % in 2023 (Githure et al., 2022). The country has five malaria endemic zones which fully cover the country (KMIS, 2021) and the zones are highland epidemic prone, lake endemic, coast endemic, seasonal and low risk. Stable transmission occurs in the highland epidemic prone, followed by the lake endemic and coastal endemic zones whereas unstable transmission occurs in seasonal and low risk zones(Ministry of Health, 1994). Most of these transmissions are caused by *Plasmodium falciparum* species (Ministry of Health, 1994) and influenced by rainfall, type of vector species, altitude, intensity of biting, accessibility to health care, type of the house, distance of human houses to vector habitats, bed net use and socioeconomic factors (Ministry of Health, 1994; Guerra et al., 2018; Bannister-Tyrrell et al., 2018). These transmissions are more in children, pregnant women, persons living with HIV/AIDS, non-Indigenous residents, and visitors (Ministry of Health, 1994). Vector control methods such as mosquito nets, indoor residual spraying (Girum et al., 2019) and early diagnosis of pregnant women using artemisinin-based combination therapy (Aregawi et al., 2014) have been implemented across the country to fight malaria (Table 4). However, malaria is a disease shaped by environment, socio-economic and political factors (Samadoulougou et al., 2014). Environment drivers affect life-history traits of vectors such as rate of development and biting rates (Castillo-Salgado et al., 2015), and socio-economic and behavior factors such as housing conditions, health infrastructure also contribute to malaria transmission (Dickinson et al., 2011; Sharma et al., 2021). Therefore, for the country to achieve its goal, the current malaria distribution trend and the interventions in place need to be understood. According to our knowledge, no studies have used Kenya malaria indicator surveys, climate data and Fuzzy overlay methods nation-wide to identify how malaria incidences and its interventions relate to environmental drivers among school aged children in Kenya. The study by (Khan et al., 2023a) used Getis-Ord Gi*local test and generalized linear model (GLM) to identify factors associated with occurrence of transmission malaria hot-spots. In addition, the data set used is different from the 2015 and 2020 Kenya MIS data sets used in this study.

**Table 4:** Summary of malaria zones and respective interventions in Kenya.

Zone	Drivers	Population affected	Interventions	Reference
Highland epidemic	Education status, employment, house structure	School aged children (<15 years)	Bed nets and malaria prophylactic antimalarials	(Essendi et al., 2019),(Kapesa et al., 2018)
Coast endemic	Age, rainfall, presence of rice fields	Children (6 months to 4 years)	Bed nets	(Kamau et al., 2020),(Bisanzio et al., 2015)
Lake endemic	Age, wealth index, farming, residence area	Children (6 months to 14 years)	Bed nets	(Bashir et al., 2019)
Seasonal	Socio-economic status	School aged children (5 months to 14 years)	Bed nets	(Gitonga et al., 2012)
Low risk	Elevation	School aged children (5 months to 14 years)	Bed nets	(Gitonga et al., 2012)

The purpose of this study is to examine spatial distribution of malaria and interventions, and how malaria incidences and interventions from the surveys relates to the environmental conditions required for malaria. The study used the 2015, and 2020 Kenya MIS with suitability analysis, and kriging interpolation methods. The results would help policymakers understand the influence of climatic and environmental factors in malaria transmission as it will be useful in planning.

## 2 Methods

### 2.1 Data

#### 2.1.1 Malaria indicator surveys (MIS)

**Fig. 1:**
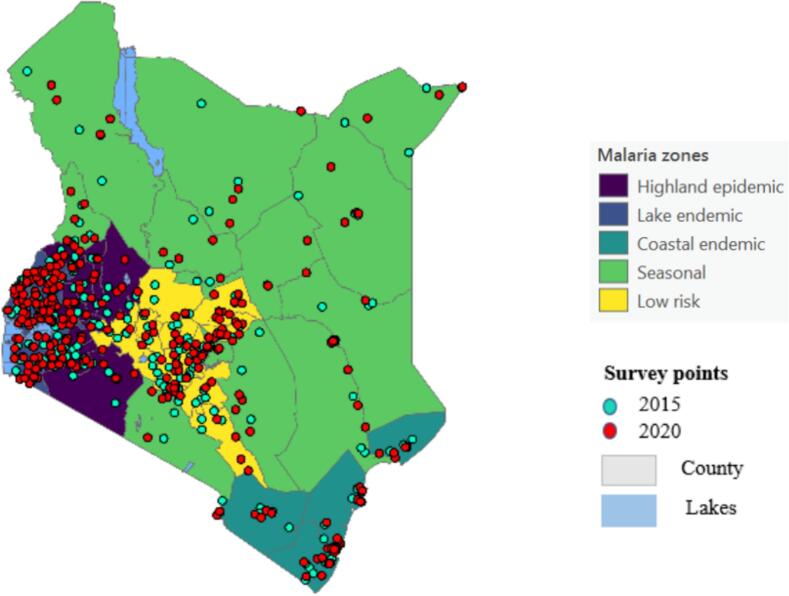
Malaria Indicator Survey locations sampled during 2015 and 2020 in five malaria zones.

Kenya has undertaken four Malaria Indicator Surveys (MIS), in 2007, 2010, 2015 and 2020 (Kenya, 2021). The 2007 MIS focused on children under the age of 5 years (Kenya Malaria Indicator Survey, 2007), and the 2010, 2015, and 2020 MIS targeted school aged children below 15 years (KMIS, 2021; KMIS, 2010; KMIS, 2016). However, the 2007 and 2010 MIS were not used in this study due to unavailability of data related to malaria and interventions as well as geographical information for each sampled location (e.g., distribution of samples Fig. 1). The Kenya MIS conducted during 2015, and 2020 were obtained from the Demographic and Health Survey (DHS) website (https://dhsprogram.com/Data/). The surveys used a two-stage stratified clustering sampling strategy to select country representative samples for accurate estimation of malaria prevalence, with a sample size of between 5000 and 30,000 households (Fig. 1). In each sampled household, children were tested for malaria using a rapid malaria diagnostic test (RDT) and the type of parasite in the blood was confirmed using a microscopy test. RDTs is one of the initial malaria tests performed during the survey as it offers the possibility to expand the arrangement of exact malaria diagnosis to the region where microscopy services are not accessible (Gaston and Ramroop, 2020). The two tests are known for estimating malaria parasite point prevalence from household surveys (Keating et al., 2009). GPS technology was used to record the geographical coordinates of each sample unit. However, in 2015, 7380 households were sampled from 246 clusters (KMIS, 2016) and 8843 households were sampled from 298 clusters in 2020 (KMIS, 2021). The surveys focused on school-aged children below 15 years old and interviewed women aged 15 to 49 years old in each household. The survey also included information about demographic (sex, age), and socio-economic (wealth index, place of residence, use of mosquito nets) questions.

#### 2.1.2 Environment data

Climate data for 2015 and 2020 was downloaded from the National Centres for Environmental Information (NCEI) website (https://www.ncei.noaa.gov/) (Brown et al., 2019) for each of the months of January 1st through to December 31st, including the months that coincided with the MIS surveys. Daily climate data for twenty-two weather stations in different counties of Kenya were obtained. Continuous climate surfaces were created by interpolating the point data using empirical Bayesian kriging (EBK) method as described in (Krivoruchko and Gribov, 2019). These data have been used by (Balasubramani et al., 2019) to identify and analyse indicators for predicting malaria incidence in Zimbabwe. Climatic variables for this study included monthly precipitation (mm) and temperature (°C).

### 2.2 Analysis

#### 2.2.1 Analysis of malaria risk in Kenya during 2015, 2020 and associated risk factors

A chi-square analysis was used to examine statistically significant risk factors associated with malaria during 2015–2020. To do so, five independent variables and malaria test results were obtained from the MIS datasets. The five variables included age, wealth index, place of residence, sex, and ownership of nets. These were reclassified into binary form using the MIS definitions for categorical values (Croft et al., 2018). For example, MIS classifies age as a continuous variable from 6 months to 14 years. In this study, age was classified as under five years for 6 months to 4 years old and above four years was classified as 5–14 years. MIS classifies wealth index as poor, poorer, poorest, middle, rich, richer, and richest. This study classified wealth index as poor if it was poor, poorer, or poorest, and rich if it was rich, richer, and richest. MIS also classifies malaria test result as positive and negative, in this study we classified positive as 1 and negative as 0. The reclassified variables (Table 5) were used to perform Pearson's Chi-square test in R for Windows 4.2.1.

**Table 5:** Malaria suitability groups and associated temperature and precipitation values summarized from (Blanford et al., 2013; Craig et al., 1999; Mordecai et al., 2013).

Reclassified group and associated risk	Temperature Range (°C)	Precipitation range (mm)
1 (Unsuitable)	< 18, > 25	<80
2	18–22	
3 (High risk)	22–25	≥80

#### 2.2.2 Spatial distribution of malaria cases

Malaria test results were obtained from the MIS datasets where Rapid Diagnostic Test (RDT) result with categorical response “Positive” or “Negative” was used to map and measure malaria prevalence in Kenya. Positive cases from each geographical zone were aggregated per county and by the presence of parasite tested. Dot density-based analysis was conducted using ArcGIS Pro version 3.0 software to map malaria cases by the type of parasite species. The map was represented by dots. For this case, 1 dot represented ≥1 positive cases. This method has been used by (Pham Minh et al., 2017) to quantify spatial data.

#### 2.2.3 Malaria suitability analysis

To examine risk of malaria and changing risks of malaria, a suitability method was used to create malaria risk maps for each month for the years 2015 and 2020. The method allocates respective ratings for attribute values in a given thematic layer between 1 (unsuitable) and 3 (most suitable) which are then merged to create a final risk map (Kioko and Blanford, 2023). To do so, the monthly average temperature and total precipitation layers were reclassified into malaria suitability layers (Table 5). Temperature rasters were reclassified into 3 categories where 1 = unsuitable; 2 = somewhat suitable; and 3 as the most suitable. The precipitation rasters were reclassified into 2 categories where precipitation greater or equal to 80 mm was assigned suitable (3) and anything below 80 mm was assigned unsuitable (1). The risk categories used for temperature and precipitation are summarized in Table 5 and are based on the host-pathogen-environment relationship of malaria as described in (Blanford et al., 2013; Craig et al., 1999), (Mordecai et al., 2013). Once each layer was reclassified, they were combined using the fuzzy overlay method (AND) to create monthly risk maps for Kenya during 2015 and 2020.

#### 2.2.4 Spatial distribution of ITNs usage

The question “Who slept under this mosquito net last night?” with a binary response of “Yes” or “No” was used to measure ITNs use among households with nets. ITNs users were aggregated by county, and proportion of ITNs users was determined by dividing the total number of households using nets by the total number of households who were interviewed for that county (ITNs usage_county, i_ = total number of households using nets for county i/total number of households interviewed for county i).

#### 2.2.5 Spatial distribution of malaria knowledge

The question “Do you know about malaria?” with a binary response of “Yes” or “No” was used to measure mother's malaria knowledge about malaria and its interventions. The proportion of mothers aware about malaria was determined by dividing the total number of households responded “yes” by the total number of households who were interviewed for that county (Malaria knowledge_county, i_ = total number of households responded “yes” for county i/total number of households interviewed for county i).

## 3 Results

### 3.1 Malaria in Kenya during 2015, 2020 and associated risk factors

The 2015 malaria survey was conducted in July and over 10,072 children were tested for malaria. Thirteen percent were found positive and 87 % were negative. Eighty six percent of the children aged 5–14 years tested positive and 14 % were negative, while 3 % of children below 5 years tested positive and 97 % were negative. Fifteen percent of children owning ITNs tested positive and 85 % were negative, while 6 % of children without nets tested positive and 94 % were negative. Seventeen percent of children from rural areas tested positive and 84 % were negative, while 7 % of children from urban areas tested positive and 93 % tested negative. Twelve percent of female children tested malaria positive and 88 % were negative, while 13 % of male children tested positive and 87 % were negative. Sixteen percent of children from poor background tested malaria positive and 84 % were negative, 15 % from medium status tested positive and 85 % tested negative, and 6 % of children from rich family tested malaria positive and 94 % were negative. Owning ITNs (***p*** **<** **0.0000)**, place of residence (***p*** **<** **0.0000)**, and wealth index (***p*** **<** **0.0000)** factors were statistically significant factors associated with malaria results at 5 % level of significance.

In 2020 the survey was conducted in November where over 11,549 children were tested for malaria. Ten percent of the children were found positive while 90 % were not. Twelve percent of the children aged 5 to 14 years old tested positive and 88 % were negative, while 7 % of the children below 5 years old tested malaria positive and 93 % were negative. Twelve percent of the children owning ITNs tested malaria positive and 88 % tested negative, while 7 % of the children without nets tested positive and 93 % tested negative. Thirteen percent of the children living in rural areas tested positive and 87 % tested negative while 4.0 % of children living urban areas tested malaria positive and 96 % tested negative. Ten percent of male children tested positive and 90 % tested negative while 11 % of female children tested positive and 90 % tested negative. Thirteen percent of children from poor family tested positive and 87 % tested negative, 11 % from middle class tested positive and 89 % tested negative, 5 % from rich background tested positive and 95 % tested negative. Age of the child (***p*** **<** **0.0000)**, ownership of ITNs (***p*** **<** **0.0000)**, place of residence (***p*** **<** **0.0000)**, and wealth index (***p*** **<** **0.0000)** were significant factors associated with malaria test results at 5 % level of significance.

### 3.2 Spatial distribution of malaria cases during 2015 and 2020

**Fig. 2:**
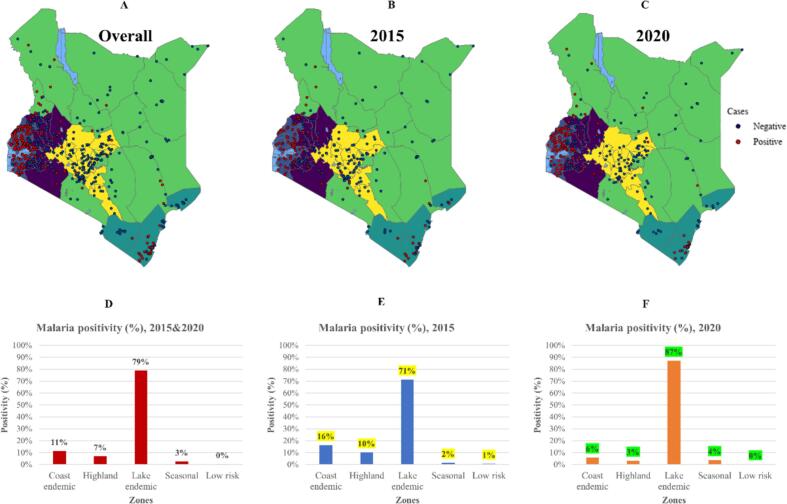
The overall (A), 2015 (B), and 2020 (C) spatial distribution of malaria positive and negative cases in each malaria zone. Graphs showing overall (D), 2015 (E), and 2020 (F) proportion of positive cases in each zone.

Overall, majority of malaria positive cases were distributed in the lake endemic zone (79 %), followed by coastal endemic (11 %), highland epidemic (7 %), seasonal (3 %), and low risk (<1 %) as shown in Fig. 2A. In 2015, most cases were in the lake endemic zone (71 %), followed by coastal endemic (16 %), highland epidemic (10 %), seasonal (2 %), and low risk (1 %) as shown in Fig. 2B. During 2020, more cases were in the lake endemic zone (87 %), followed by coastal endemic (6 %), seasonal (4 %), highland epidemic (3 %), and low risk (<1 %), see Fig. 2C. Malaria cases has reduced by 10 % in the coastal endemic zone, reduced by 7 % in highland epidemic zone, increased by 16 % in the lake endemic zone, increased by 2 % in seasonal zone and reduced by 1 % in the low-risk zone (see Fig. 2D-F).

### 3.3 Spatial distribution of malaria interventions during 2015 and 2020

**Fig. 3:**
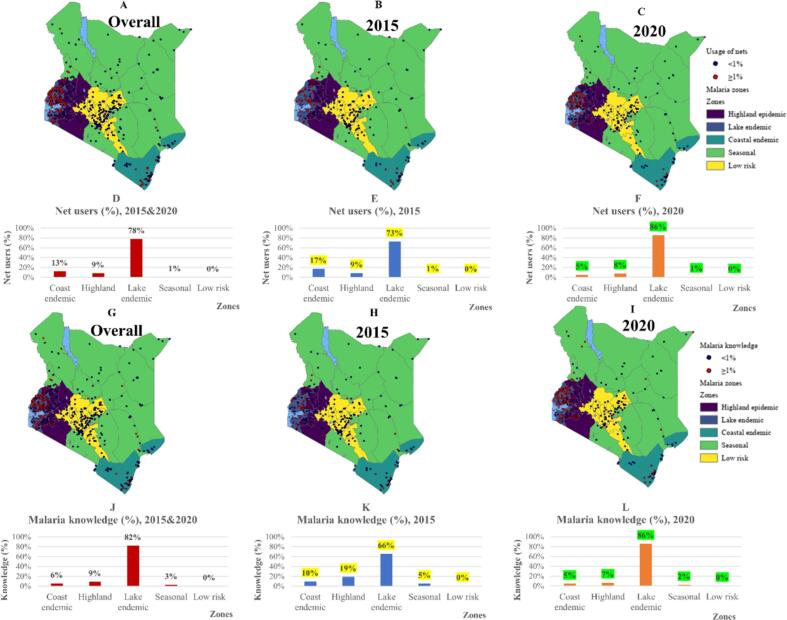
The overall (A), 2015 (B), 2020 (C) spatial distribution of ITNs usage and graphs (D—F) showing proportion of ITNs users in each malaria zones. Overall (G), 2015 (H), 2020 (I) spatial distribution of malaria knowledge and graphs (J-L) showing proportion of mothers with malaria knowledge in each zone.

Overall, majority of ITNs users are distributed in the lake endemic zone (77 %), followed by coastal endemic (13 %), highland epidemic (9 %), seasonal (1 %), and low risk (<1 %) as shown in Fig. 3A. In 2015, most net users were in the lake endemic zone (73 %), followed by coastal endemic (17 %), highland epidemic (9 %), seasonal (1 %), and low risk (<1 %), see Fig. 3B. During 2020, more ITNs users were in the lake endemic zone (86 %), followed by highland epidemic (8 %), coastal endemic (5 %), seasonal (1 %), and low risk (<1 %) as shown in Fig. 3C. The number of ITNs users has reduced by 12 % in the coastal endemic zone, reduced by 1 % in highland epidemic zone, increased by 13 % in the lake endemic zone, and remained the same in seasonal and low-risk zone (Fig. 3D-F).

Majority of mothers with malaria knowledge are distributed in the lake endemic zone (82 %), followed by highland epidemic (9 %), coastal endemic (6 %), seasonal (3 %), and low risk (<1 %), see Fig. 3G. In 2015, mothers with malaria knowledge were more in the lake endemic zone (66 %), followed by highland epidemic (19 %), coastal endemic (10 %), seasonal zone (5 %), and low risk (<1 %), see Fig. 3H. During 2020, more mothers were in the lake endemic zone (86 %), followed by highland epidemic (7 %), coastal endemic (5 %), seasonal (2 %), and low risk (<1 %) as shown in Fig. 3I. The number of mothers with malaria knowledge has reduced by 5 % in the coastal endemic zone, reduced by 12 % in highland epidemic zone, increased by 20 % in the lake endemic zone, reduced by 3 % in seasonal zone and remained the same in low-risk zone (Fig. 3J-L).

### 3.4 Precipitation and temperature 2015 and 2020

**Fig. 4:**
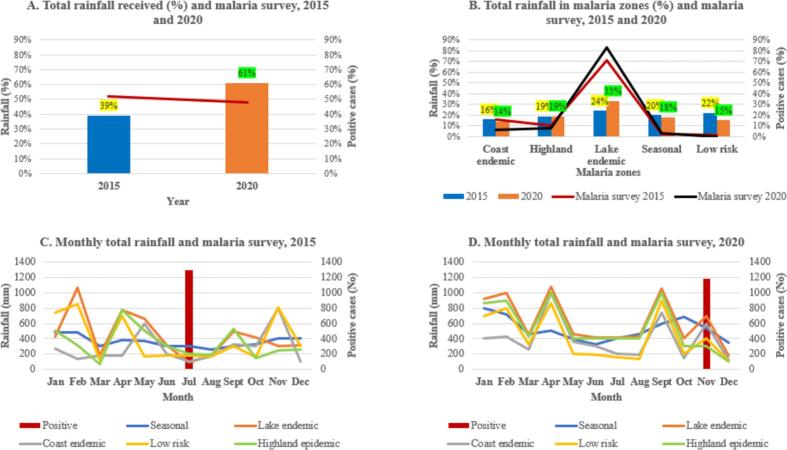
Annual total precipitation, 2015 and 2020 (A), annual total precipitation in each malaria zones (B), total monthly precipitation (mm), 2015 (C) and 2020 (D). Red bar represents total positive malaria survey data during July 2015 and November 2020. (For interpretation of the references to colour in this figure legend, the reader is referred to the web version of this article.)

The total amount of rainfall received during 2015 was 22 % less than the amount of rainfall received in 2020. This means 2020 was a wetter year than 2015, and based on malaria survey more positive cases were recorded in 2015 compared to 2020 (Fig. 4A). Overall, lake endemic was wetter, followed by highland and seasonal zones, low risk and coastal endemic. Highland epidemic zone received the same amount of rainfall (19 %) in 2015 and 2020, coast endemic zone was less wet in 2020 by 2 %, lake endemic zone was wetter in 2020 by 9 % and had majority of the malaria cases, seasonal zone was wetter in 2015 by 2 % as well as low-risk zone by 7 %. (Fig. 4B).

Based on monthly rainfall, in 2015, low risk zone was wetter in January, lake endemic zone in February, seasonal zone in March, lake endemic and highland epidemic in April, lake endemic in May, seasonal, highland and lake endemic zones in June, seasonal zone in July and August when MIS survey was conducted, highland epidemic and lake endemic in September, lake endemic in October, low risk and coast endemic in November, and seasonal zone in December (Fig. 4C). During 2020 lake endemic was wetter in January through September, seasonal zone in October, lake endemic in November when MIS survey was conducted, and seasonal zone in December (Fig. 4D).

### 3.5 Temperature during 2015 and 2020

**Fig. 5:**
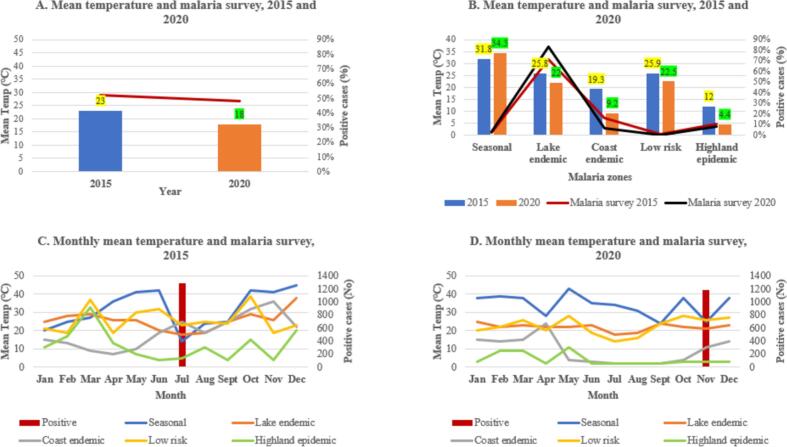
Annual mean temperature, 2015 and 2020 (A), annual mean temperature in each malaria zones (B), monthly mean temperature in °C, 2015 (C) and 2020 (D). The red bar represents total positive malaria survey data during July 2015 and November 2020. (For interpretation of the references to colour in this figure legend, the reader is referred to the web version of this article.)

The annual mean temperature received in 2015 was higher by 5 % compared to 2020. This implies that 2015 was hotter than 2020, and according to malaria survey, the same year had more malaria cases (Fig. 5A). Overall, seasonal zone was hotter, followed by low risk, lake endemic, coastal endemic and highland epidemic. Seasonal zone was hotter in 2020 by 2.5 °C, while lake endemic zone, coast endemic zone, low risk zone and highland epidemic zone were less hotter in 2020 by 3.8 °C, 10.1 °C, 3.4 °C, and 7.6 °C respectively (Fig. 5B).

According to monthly mean temperature, in 2015, lake endemic zone was hotter in January and February, low risk zone in March, seasonal in April through June, coast endemic in July when MIS survey was conducted, low risk in August and September, and seasonal in October through December (Fig. 5C). In 2020, seasonal zone was hotter in January through December and when MIS was conducted (Fig. 5D).

### 3.6 Malaria risk maps and malaria zones, 2015 and 2020

**Fig. 6:**
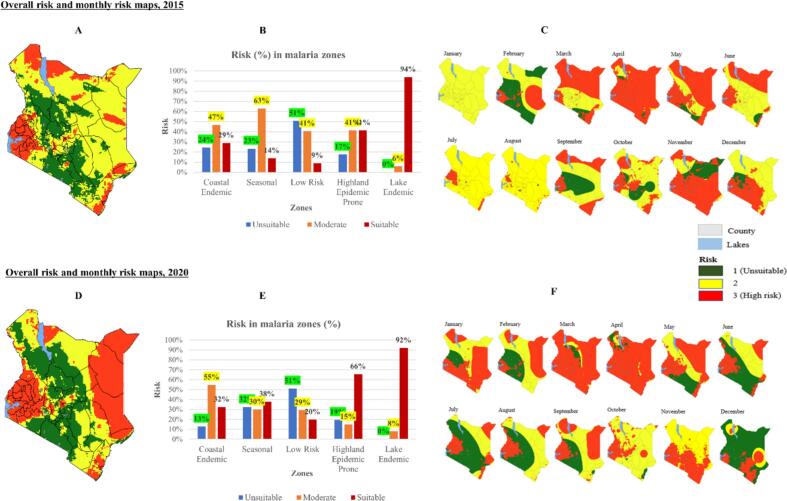
The overall risk, 2015 (A) and 2020 (D), and monthly risk maps based on temperature and precipitation 2015 (C) and 2020 (F). The graphs show % of the risk covered in each malaria zone during 2015 (B) and 2020 (E).

Based on temperature and precipitation, in 2015, areas around Lake Victoria, Lake Turkana, and coastal regions are suitable (red shades) for malaria transmission, eastern areas are moderate (yellow), central areas are unsuitable (green) for transmission (Fig. 6A). Forty-seven percent of coastal endemic zone is moderate for transmission, 29 % suitable, and 24 % is unsuitable. Sixty-three percent of seasonal zone is moderate for transmission, 23 % unsuitable, and 14 % suitable. Fifty-one percent of low risk zone is unsuitable for transmission, 41 % is moderate, and 9 % is suitable. Forty-one percent of highland epidemic zone is suitable for transmission, 41 % is moderate and 17 % is unsuitable. Ninety-four percent of lake endemic zone is suitable for transmission, 6 % moderate and 0 % unsuitable (Fig. 6B).

In 2020, counties surrounding Lake Victoria, areas along the east of Lake Victoria, eastern Kenya, and coastal areas are suitable for transmission while central areas are unsuitable and moderate (Fig. 6D). Fifty-five percent of coastal endemic zone is moderate for transmission, 32 % suitable, and 13 % unsuitable. Thirty-eight percent of seasonal zone is suitable for transmission, 32 % unsuitable, and 30 % moderate. Fifty-one percent of low risk zone is unsuitable for transmission, 29 % moderate and 2015 suitable. Sixty-six percent of highland epidemic zone is suitable for transmission, 19 % unsuitable, and 15 % moderate. Ninety-two percent of lake endemic zone is suitable for transmission, 8 % moderate and 0 % unsuitable (Fig. 6E).

The 2015 monthly risk maps show heterogeneity of transmission across Kenya, with high risk associated with areas surrounding Lake Victoria (February–December), areas along Lake Turkana (March–June, August–September) and low risk in counties surrounding Nairobi (February, September, October) as shown in Fig. 6C. In 2020, high risk is associated with areas around Lake Victoria (January through December), counties in eastern Kenya (January–May, September) and low risk in central Kenya (February, June–September, December) and counties in northern Kenya (June, July, December), see Fig. 6F.

In relation to malaria survey, counties surrounding Lake Victoria region and Indian Ocean were suitable for transmission when 2015 (July) and 2020 (November) MIS was conducted, and the rest were moderate for malaria transmission.

### 3.7 Malaria incidences, net usage, and malaria knowledge in malaria suitable zones 2015 & 2020

**Fig. 7:**
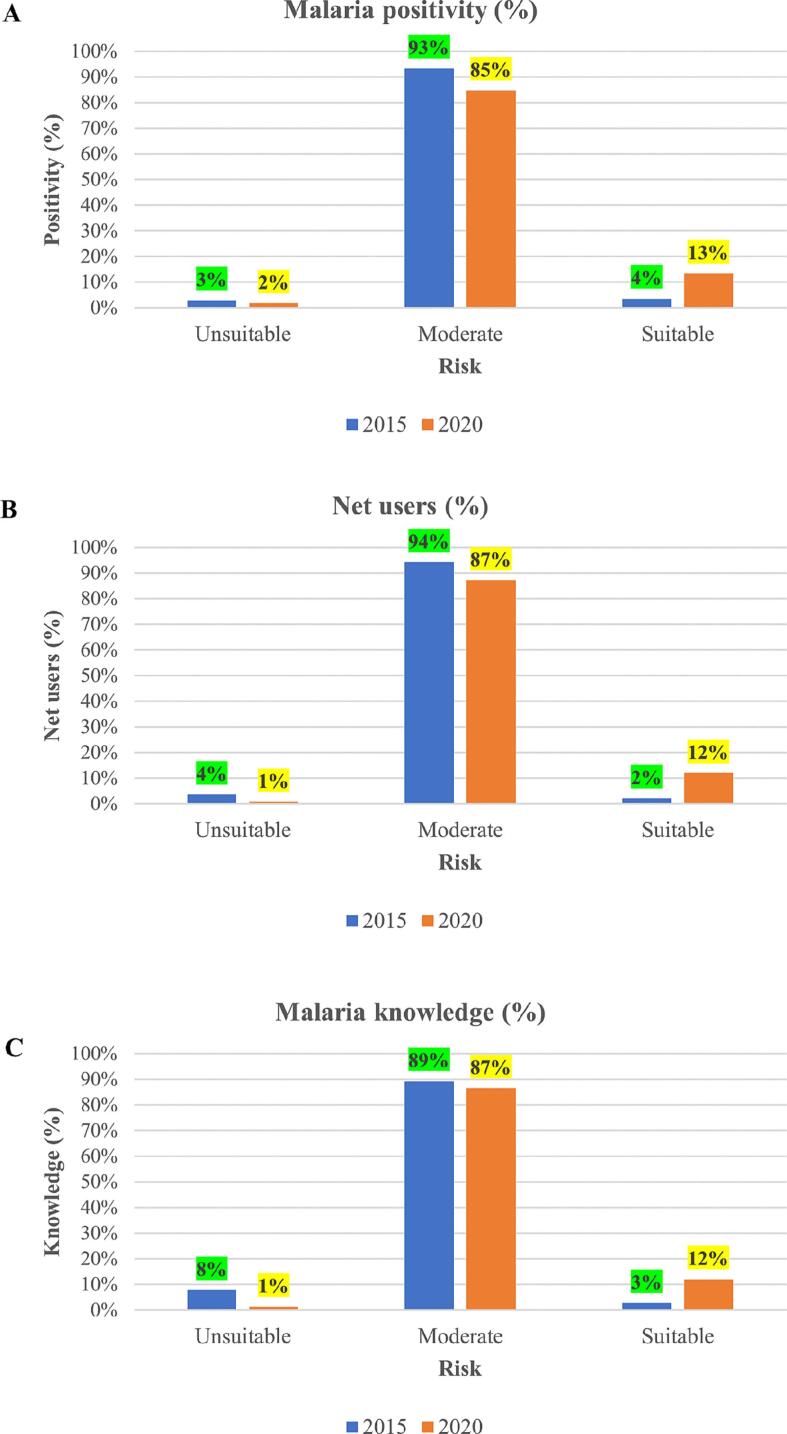
The graphs show malaria incidences (A), ITN usage (B), and malaria knowledge (C) in malaria suitable zones, 2015 and 2020.

Overall, majority of malaria cases are found in moderate areas, followed by suitable and unsuitable. In 2015, 93 % of malaria positive cases are found in moderate areas, 4 % in suitable and 3 % in unsuitable. During 2020, 85 % of malaria positive cases are found in moderate areas, 13 % suitable and 2 % unsuitable. Malaria positive cases has reduced by 8 % in moderate, reduced by 1 % in unsuitable, and increased by 9 % in suitable areas (Fig. 7A).

Overall, more ITNs users reside in moderate areas, followed by suitable and unsuitable areas. In 2015, 94 % of net users are found in moderate, 4 % in unsuitable, and 2 % in suitable areas. In 2020, 87 % of ITNs users reside in moderate areas, 12 % in suitable areas and 1 % percent in unsuitable areas. From the results, the proportion of net users in moderate areas has reduced by 7 %, increased by 10 % in suitable areas, and reduced by 3 % in unsuitable areas (Fig. 7B).

Overall, mothers with malaria knowledge about malaria are more in moderate areas, followed by suitable and unsuitable. In 2015, 89 % with malaria knowledge are found in moderate areas, 8 % in unsuitable areas, and 3 % in suitable areas. In 2020, 87 % reside in moderate areas, 12 % suitable areas and 1 % unsuitable areas. The findings show that mothers with knowledge about malaria has dropped by 2 % in moderate areas, increased by 9 % in suitable areas, and reduced by 7 % in unsuitable areas (Fig. 7C).

## 4 Discussion and conclusion

Despite the availability of interventions, malaria remains a public threat in Kenya causing deaths among children, especially in Western Kenya (Hollowell et al., 2023). With changing climate, suitable areas for malaria transmission are expanding across the country. Malaria cases are increasing in areas which have been known as less endemic, for example, Northern Kenya region, and cases are declining in endemic areas, for example, coastal Kenya (Snow et al., 2015). To better control and eliminate malaria it is crucial to understand where malaria and interventions are spatially distributed. If the risk is known and people are aware about the risk and how to respond, may be malaria can be eliminated. The strong connection between malaria transmission and changes in environmental conditions, especially temperature and rainfall patterns, which are the main drivers of malaria transmission (Mordecai et al., 2017, Mordecai et al., 2019) makes it easier to conduct seasonal analysis using daily temperatures and rainfall to monitor within seasonal risk. In this work, we examined the spatial distribution, and how malaria incidences and interventions from the survey relates to the environmental conditions required for malaria. The study examined the 2015, and 2020 Kenya MIS and used suitability analysis, and kriging interpolation methods to map malaria risk areas, demonstrating how these methods and data are useful for capturing areas with malaria risk. The results are useful for monitoring risk and can help policy makers understand the influence of climatic and environmental factors in malaria transmission as well as for planning intervention strategies.

MIS is a nationwide malaria survey conducted every 5 years and provides information about malaria and interventions in 5 different malaria zones. In this case, we used the two latest MIS (2015 and 2020) to measure the progress of malaria and interventions within zones. We found that malaria cases have reduced in coastal endemic, highland epidemic, and low-risk zone and increased in lake endemic zone and seasonal zone (Fig. 2A-F). For interventions, bed net usage and malaria knowledge has reduced in coastal endemic and highland endemic and increased in lake endemic. ITNs usage remained the same in seasonal and low-risk zone (Fig. 3A-F) and mothers with knowledge about malaria reduced in seasonal zone and remained the same in low-risk zone (Fig. 3G-L). The result implies that despite a high usage of ITNs and being aware about malaria, malaria cases are still increasing in lake endemic zone. The unexpected results of declining malaria cases, usage of ITNs and malaria knowledge in coastal endemic zone, which is known as a malaria hotspot (Id and Gichuki, 2023) could be due to climate change in the region which has led to emerging of new arboviruses such as dengue and chikungunya (Mordecai et al., 2020; Ngoi et al., 2016). In addition, the seasonal zone may be the next region with more cases since knowledge about malaria has declined as well as use of bed nets.

Since the Malaria surveys were conducted at different times of the year (e.g. 2015 MIS was conducted in July and 2020 MIS in November), we examined how malaria risk changes throughout the year in Kenya by developing monthly risk maps (Fig. 6C&F). The maps highlighted spatial variations of malaria across Kenya identifying areas surrounding Lake Victoria, Lake Turkana, and the Indian Ocean as the most suitable for malaria transmission. These areas have previously been found to be high malaria prevalence areas (Macharia et al., 2018b; Alegana et al., 2021; Gopal et al., 2019). Examining the relationship between malaria survey and risk, lake endemic zone was found suitable for transmission and with higher incidences of malaria and low-risk zone as unsuitable with few malaria cases. This may imply that lake endemic zone still remains a hotspot for transmission as found in other studies (Otambo et al., 2022; Khan et al., 2023b; Alegana et al., 2021). Also, the findings highlighted an increase of malaria cases, ITNs usage, and malaria knowledge in suitable areas as well as a reduction of the cases, ITNs usage, and malaria knowledge in moderate and unsuitable areas.

Although the method presented here are useful, there are several limitations of this study. First, MIS are conducted every 5 years during rainy season. Therefore, malaria incidence may not be representative for all areas and during the highest transmission period. Second, socio-demographic, and economic factors (Bashir et al., 2019), accessibility to health care facilities (Hasyim et al., 2019), and human mobility (Guerra et al., 2019) are not considered, although these factors have been found to be associated with malaria risk. Further work will examine the underlying causes of the spatial variation in malaria incidence in Kenya and determine what interventions measures may be needed to help further reduce malaria in suitable areas.

In conclusion, the result from this study demonstrates that daily temperature and rainfall can be used to create real time local seasonal risk map, thus enabling for the monitoring of seasonal malaria risk. The methods are simple and easy to use; the data requirements are minimal using information that is readily available. Not only can these outputs be used to assess where the risks are but also identify intervention gaps and need.

## CRediT authorship contribution statement

**Caroline Kioko:** Writing – review & editing, Writing – original draft, Visualization, Methodology, Investigation, Conceptualization. **Justine Blanford:** Writing – review & editing, Supervision, Conceptualization.

## Declaration of competing interest

None.
